# Long-Term Prognosis of Endodontic Microsurgery—A Systematic Review and Meta-Analysis

**DOI:** 10.3390/medicina56090447

**Published:** 2020-09-03

**Authors:** Diogo Pinto, Andréa Marques, Joana F. Pereira, Paulo J. Palma, João Miguel Santos

**Affiliations:** 1 Department of Dentistry, Faculty of Medicine, University of Coimbra, 3000-075 Coimbra, Portugal; diogodentuc@gmail.com; 2 Rheumatology Department, Centro Hospitalar e Universitário de Coimbra, 3000-075 Coimbra, Portugal; andreamarques23@esenfc.pt; 3 Health Sciences Research Unit: Nursing, UICISA-E, 3000-075 Coimbra, Portugal; 4 Institute of Endodontics, Faculty of Medicine, University of Coimbra, 3000-075 Coimbra, Portugal; joaninhafpereira@gmail.com (J.F.P.); ppalma@uc.pt (P.J.P.); 5 Center for Innovation and Research in Oral Sciences (CIROS), Faculty of Medicine, University of Coimbra, 3000-075 Coimbra, Portugal

**Keywords:** endodontic microsurgery, endoscope, root canal therapy, systematic review, surgical endodontic retreatment, survival rate, treatment outcome

## Abstract

*Background and objectives:* The long-term outcome of endodontic microsurgery (EMS) performed on root-filled teeth affected by post-treatment apical periodontitis (AP) has been a matter of debate, re-launched by the introduction of novel root-end filling materials which have been proven to improve the short-term outcome of EMS. The purpose of this systematic review and meta-analysis is to evaluate the clinical and radiographic long-term outcome of endodontic microsurgery in teeth diagnosed with secondary AP through radiographic evaluation. *Materials and Methods:* This systematic review and meta-analysis followed the Preferred Reporting Items for Systematic Reviews and Meta-Analyses (PRISMA) guidelines. Inclusion and exclusion criteria were defined a priori to select the best longitudinal evidence. Only randomized clinical trials (RCT) and prospective clinical studies (PCS), with a follow-up ≥ 2-year, and exhibiting well-established clinical and radiographic outcome criteria, were selected. *Results:* A total of 573 articles were obtained, from which 10 fulfill inclusion criteria: 6 PCS and 4 RCT. Meta-analysis showed a pooled proportion of success rate of 91.3%, from an overall amount of 453 treated teeth included in RCT; from overall 839 included teeth in PCS, a pooled success rate of 78.4% was observed, with the follow-up time ranging from 2 to 13-years. Survival rate outcomes varied from 79 to 100% for the same follow-up period. Five prognostic factors with influence on the outcome were disclosed: smoking habits, tooth location and type, absence/presence of dentinal defects, interproximal bone level, and root-end filling material. *Conclusions:* High success rates and predictable results can be expected when EMS is performed by trained endodontists, allowing good prognosis and preservation of teeth affected by secondary AP.

## 1. Introduction

The main etiology of post-treatment apical periodontitis (AP) is persistent or secondary intraradicular infection, and the preferable treatment option to manage this clinical situation is nonsurgical endodontic retreatment (NSER) [1]. If NSER is supposed to be technically challenging, associated with the risk of complications or refusal by the patient, surgical endodontic retreatment (SER) is an alternative approach to preserve the tooth and avoid the extraction [2,3]. Moreover, infection in apically unreachable areas, extraradicular infection, foreign body reactions, or radicular cysts are other possible causes of AP, which can be better addressed clinically with SER [4,5].

Endodontic microsurgery (EMS) is a current SER approach characterized by modern microsurgical techniques that integrate the use of an operation microscope [6] or an endoscope [7], root-end cavity preparation with ultrasonic tips, and more biocompatible root-end filling material [3,8].

Over the years, the use of magnification devices has become an increasingly common practice in dentistry [9]. During the endodontic microsurgery procedure, the operator can easily identify root apices and anatomical details, such as isthmuses, root microfractures, canal fins, and lateral canals. Combined with the microscope or endoscope, the use of ultrasonic tips enables a conservative surgical approach, for the reason that this technology allows the operator better control during the procedure and decreases the risk of perforation by increasing the capacity to remain localized in the center of the canal compared to the microhandpiece [10]. With the aim of preventing the outgrowth of bacteria and promote periapical tissue healing [11], an ideal root-end filling material must have biocompatibility, dimensional stability [6], and resistance to resorption [11]. Therefore, it should be bactericidal, bacteriostatic, easy to manage, and offer an exceptional seal capacity [6,11].

The outcome evaluation of EMS is based on the combination of specific clinical and radiographic healing criteria. Several parameters are required to be evaluated to establish clinical success, such as existence or absence of signs and/or symptoms, tenderness to percussion or palpation, mobility, and function. In addition, sinus tract formation or periodontal pocket development must be examined [12]. Currently, the radiographic outcome classification defined by Rud et al. [13] and Molven et al. [14] is known to be broadly accepted for clinical practice due to its solid correlation between radiographic and histologic findings from 120 teeth [13] and its high interobserver agreement after isolated examinations [3,14]. Therefore, this classification is divided into four categories (Table S1): complete healing (re-formation of the lamina dura), incomplete healing (scar tissue), uncertain healing, and unsatisfactory healing [12].

Furthermore, the use of cone-beam computed tomography (CBCT) can be, in the next future, an excellent alternative to evaluate long-term healing of EMS in three dimensions [2], as it has been demonstrated to be more sensitive and specific than periapical radiographs in assessing periapical radiolucencies [15]. In line with this, the “Penn 3D criteria” of assessment emerge with three categories: complete healing, limited healing, and unsatisfactory healing [16]. Kim et al. [10] also developed a classification with the purpose of making a prediction of the probability of endodontic microsurgical success depending upon the pre-existing condition of the tooth.

The prognosis of EMS can be influenced by several variables, for instance: differences in the procedures and materials; clinical and radiographic evaluation; patient-related factors (demography and systemic condition); type, number, and location of the tooth involved; the quality of earlier root canal treatment or retreatment, and the type of coronal restorations. It is difficult to make a direct comparison between different studies about this subject when there is an obvious heterogeneity for these variables and for the evaluation of the treatment success and failure rate among the studies [9,17].

For these reasons, there is a need to overcome the heterogeneity of evidence about the prognosis of EMS [1] through the differentiation of the studies according to their methodological rigor [18], with the aim of decreasing the large variety of reported outcomes [1]. Furthermore, the development of this review aims to achieve a more reliable outcome result through the search of studies with a high level of external validity of the results, particularly concerning the follow-up period evaluated and the conditions in which EMS was performed. In this way, we expect to solve some limitations of previous reviews on the short-term outcome of EMS [19,20,21] and disclose more reliable and pragmatic evidence to use in a private practice clinical context.

Kang et al. [20] developed a systematic review and meta-analysis with the purpose of evaluating and comparing the clinical and radiographic outcomes of nonsurgical endodontic retreatment and endodontic microsurgery and presented 92% of the overall pooled success rate for EMS. Nonetheless, this review [20] included studies with a minimum sample size of 20 teeth, which may not be enough to reach clinically meaningful results [18]. Moreover, a systematic review and meta-analysis developed by Seltzer et al. [21] concluded that endodontic microsurgery showed significantly better prognosis than traditional root-end surgery (TRS), with a 94% success rate to treat apical periodontitis through EMS. However, such a conclusion is based on studies with a minimum follow-up period of 6-months [22].

During the last two decades, many studies have reported on the outcome of EMS, notwithstanding only short-term (≤2-years) follow-up [23,24,25,26,27,28]. Short-term observation may overestimate the prognosis because 5 to 25% of teeth assessed as healed at the short-term have been reported to revert healing when observed at 3-years or longer after EMS [7,29,30,31]. Moreover, the proportion of cases assessed as “uncertain healing” at a 1-year follow-up that will progress to “complete healing” when assessed at 5-years is variable and dependent upon the root-end filling material [7,31,32]. Therefore, reviewing selected studies to assess a high-level of evidence on the long-term healing and survival of teeth submitted to EMS is needed to clarify this gap in knowledge. Evidence on the long-term outcome of EMS is extremely important to give reliable information to treatment providers to have a sound rationale when weighing alternative treatment options with their patients [7]. This clinical evidence is expected to improve the identification of predictive factors with an impact on the prognosis and possibly will have a better intertwining with patient-centered outcomes.

The purpose of this systematic review and meta-analysis is to evaluate the clinical and radiographic long-term outcome of endodontic microsurgery in teeth diagnosed with secondary AP through radiographic evaluation.

## 2. Materials and Methods

Before the literature search, a specialized framework based on evidence was used, known as PICO question (population, intervention, comparison, outcome). This question was formulated as follows: “What is the long-term clinical and radiographic outcome of endodontic microsurgery (EMS) in teeth diagnosed with secondary AP through radiographic evaluation?”.

A preliminary search of the JBI Database of Systematic Reviews and Implementation Reports, the Cochrane Database of Systematic Reviews, PROSPERO, and MEDLINE revealed that currently, there is no other review (published or in progress) on this topic. Manuscript preparation complied with PRISMA guidelines.

### 2.1. Searching Criteria

The inclusion and exclusion criteria allowed making a rigorous selection of all the clinical studies that evaluated the clinical and radiographic outcomes after endodontic microsurgery.

Inclusion criteria:

1. Clinical studies in humans.
2. Publication from January 1990 to May 2020 (monthly updated since October 2019).
3. Randomized clinical trials (RCTs) on EMS.
4. Prospective clinical studies (PCSs) on EMS.
5. A minimum follow-up period of 2-years.
6. Teeth with indication to perform EMS (periapical lesion, secondary apical periodontitis, extrusion of root canal filling material resulting from primary endodontic treatment, or persistent extra-radicular infection).
7. The treatment procedure pursued the modern technique using magnification devices (microscope and endoscope) and ultrasonic root-end preparation.
8. Well-established clinical and radiographic outcome criteria (defined by Rud et al. [13] and Molven et al. [14]).
9. The success rate for EMS was given.

Exclusion criteria:

1. Studies that included patients aged under 18-years.
2. Retrospective clinical trials, cases of series, or reviews on EMS.
3. Studies with samples of teeth that were subject to root resections and amputations, or that present root perforations or fractures.
4. The procedure was not described with clarity or did not sustain the modern technique portrayed above.
5. The follow-up period was less than 2-years.
6. Studies in which the follow-up only included periapical radiography or clinical evaluation.
7. Studies that did not assess the outcome of apical microsurgery using specific clinical and radiographic criteria.
8. The success rate for EMS was not given.

### 2.2. Searching Method

An initial search, limited to PubMed has been undertaken to identify articles on this topic, followed by an analysis of the text words contained in the titles or/and abstracts, and of the index terms used to describe these articles. This informed the development of a search strategy, including identified keywords and index terms, which were tailored for each information source. After that, two electronic databases were searched: *Pubmed* (Supplementary S1) and *The Cochrane Library* (Supplementary S2).

The Medical Subject Headings (MeSH) terms used were: “periapical diseases”; “root canal therapy”; “apicoectomy”; “retreatment”; “microsurgery”; “treatment outcome”; and “Retrograde Obturation”. Additionally, the following terms were applied: “root-end filling”; “surgical endodontic retreatment”; “apical surgery”; “periapical surgery”; “retrograde surgery”; “endodontic surgery”; “root-end surgery”; “root-end cavity preparation”; “periradicular surgery”; “root-end resection”; “apicectomy”; “radiographic outcome”; “success rate”; and “radiographic success rate”.

### 2.3. Study Selection

The selection of the studies ended in May of 2020. All resulting articles were separately scanned by two reviewers (JMS, DP). Following the search, all identified citations were uploaded into Mendeley Desktop 1.19.4, and duplicates were removed.

After that, this process was followed. In the first step, the titles were read to exclude articles that did not gather the criteria for abstract assessment. If there were distinct opinions between the reviewers, the article was selected for abstract evaluation. In the second step, the reading of the abstracts of the chosen studies was carried out with the aim of selecting the articles according to the inclusion and exclusion criteria previously determined. If there were distinct opinions between the reviewers, the article was selected for full-text review. Finally, in the third step, the selected articles were fully read to select only those that met all the inclusion criteria described above. Communication with some authors was attempted to access relevant supplementary data to avoid any kind of assumption.

### 2.4. Data Extraction

During the data extraction process, an excel table was made up containing the following topics: study type; sample size; number of re-surgery or orthogonal retreatment cases; clinical and radiographic criteria; clinical and radiographic success rates; recall rate; follow-up period; technique and material employed; and finally, main results, limitations, and conclusions of the study.

### 2.5. Quality Assessment

Two Cochrane risk-of-bias assessment tools were used. The RoB2 tool was applied to randomized controlled trials, and the ROBINS-I tool was applied to non-randomized studies (https://www.riskofbias.info). The assessments were performed independently by two authors (JMS, DP). Eligible studies were assessed for methodological validity prior to inclusion in the review.

### 2.6. Meta-Analysis

Studies were pooled in a statistical meta-analysis of proportions with Freeman-Tukey double arcsine transformation [33] using OpenMeta[analyst]. Heterogeneity was assessed statistically using the standard chi-squared and I^2^ tests. Statistical analyses were then performed using random-effects models only in the presence of moderate to high heterogeneity (I^2^ > 50%), and in their absence, fixed-effects models were used instead [34]. Where statistical pooling was not possible, the findings were presented in a narrative form, including tables and figures, to aid in data presentation where appropriate.

## 3. Results

**Figure 1:**
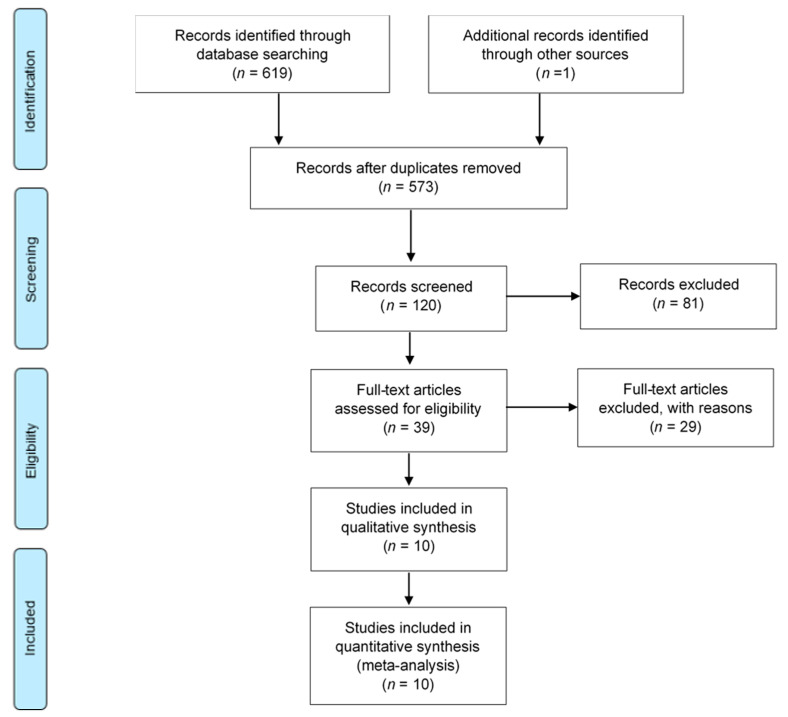
Flowchart of the article selection method according to The PRISMA Statement [35]. PRISMA: Preferred Reporting Items for Systematic Reviews and Meta-Analyses.

The article selection method of the electronic databases search is presented in Figure 1. There were 573 articles identified after database searching. Cross-references of these articles revealed one additional article that was relevant. A total of 120 records were selected after title reading was performed. Following abstract and full-text reading, a total of 110 articles were excluded according to the exclusion criteria previously established. The reasons for the exclusion are shown in Table S2. The most frequent causes of exclusion were an insufficient follow-up period (44 articles) and an unwanted study design (31 articles).

There were 10 articles that met all the inclusion criteria after full-text assessment and were subject matter to data extraction, methodologic quality assessment, and data synthesis and analysis. Table 1 summarizes information concerning the included studies in the systematic review.

Of the 10 included studies, 6 PCSs [2,7,32,35,36,37] and 4 RCTs [9,11,38,39] were selected. The minimum sample size amongst all studies was 87 teeth [35], while the maximum was 339 treated teeth [32]. Concerning the follow-up time, the minimum follow-up period was 2-years [9,36,39], and the maximum was 10 to 13 years [35]. Different types of root-end filling materials were used in the evaluated procedure, such as Mineral Trioxide Aggregate (MTA) [2,7,11,32,36,37,38,39] and Super ethoxybenzoic acid (SuperEBA) cements [7,32]. Regarding the recall rate of the studies, the lowest was 59% [39], while the highest was 89% [9]. 

All studies [2,7,9,11,32,35,36,37,38,39] applied the classification defined by Rud et al. [13] and Molven et al. [14] for outcome assessment. The overall success rate ranged from 69 [37] to 93% [38]. However, numerous potential prognostic factors were assessed during each trial (Table 2) to evaluate its influence on the outcome of EMS through statistical analysis. 

The potential prognostic factors fall into three groups: patient-related (i.e., age, sex [2,7,32,35,36,37,38]; smoking and alcohol habits [7,35]), tooth-related (clinical signs/symptoms [7], tooth location and type [2,7,9,32,35,36,37,38], previous nonsurgical [36] or surgical endodontic treatment [2,7,32,35,36], size [7,35,36] and histopathology [36] of periapical lesions, quality of root canal filling [7,36], absence/presence of a post [7,32,36,37], lesion type (A–F) [38], interproximal bone level [7], absence/presence of dentinal defect [37]), and treatment-related (i.e., type of magnification device (microscope vs. endoscope) [9], antibiotic prescription [7,35,36], root-end filling material [2,7,11,32,37,38,39], postoperative healing course [36,39]).

Statistically significant differences were found for five potential prognostic factors evaluated among the studies. Truschnegg et al., 2020 [35] showed a lower success rate in smokers (33.3%) when compared to non-smoking patients (80.4%). Tawil et al., 2015 [37], was the only study that evaluated the effect of root dentinal defects in the EMS outcome; the success rate was lower for the group of teeth with dentinal defects (31.5%) compared with the group of intact teeth (97.3%). With regard to the tooth type factor, von Arx et al., 2019 [2] showed a higher success rate for maxillary molars (95.2%) compared to maxillary premolars (66.7%). Nevertheless, no other study reported significant differences regarding this factor [7,9,11,32,35,36,37,38,39]. von Arx et al., 2012 [7] reported significant difference concerning the interproximal bone level of the tooth, showing a higher success rate when the mesial and distal interproximal bone level was ≤3 mm from the cementoenamel junction or the restoration margin of the tooth. Finally, with regard to the root-end filling material, von Arx et al., 2014 [32], reported a higher success rate for MTA (92.5%) compared with COMP (76.6%). Moreover, in another study [7], the same author also found statistically significant differences between the MTA group (86.4%) and the SuperEBA group (67.3%), with a higher success rate for MTA. However, Kim et al., 2016 [11], and Tawil et al., 2015 [37], did not report significant differences in the outcome when MTA and SuperEBA were compared.

The lowest survival rate report was 79% in a study with a follow-up period ranging from 10 to 13 years [35], and five included studies [9,36,37,38,39] reported a 100% survival rate after 2 to 10 years follow-up.

**Figure 2:**
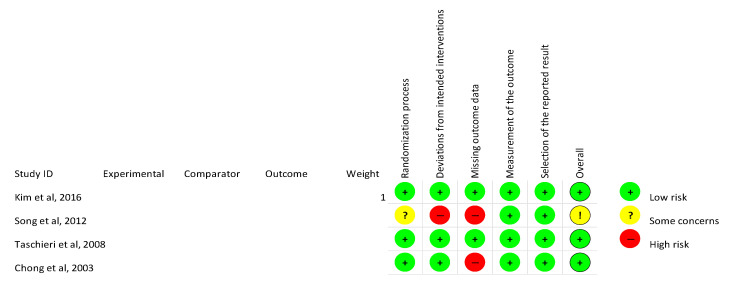
Risk of bias summary of the included randomized clinical trials (RCTs).

All 4 RCTs were subject to quality assessment through the risk of bias evaluation according to the RoB2 tool (“Risk of bias tool for randomized trials”), as recommended by Cochrane [40]. All the evaluated domains are shown in Figure 2. The overall risk of bias was low in three studies [9,11,39] and showed some concerns in the study performed by Song et al., 2012 [38].

The six remaining PCSs were subject to quality assessment through the risk of bias evaluation according to the tool ROBINS-I (“Risk of bias in non-randomized studies–of Interventions”), as proposed by Cochrane [41]. The scores are summarized in Table 3. The pre-intervention bias was shown to be mainly low risk. At the intervention, a low risk of bias was revealed among all studies. Finally, in the post-intervention domain, the risk of bias varied into low to moderate, particularly concerning the measuring outcomes.

Meta-Analysis

**Figure 3:**
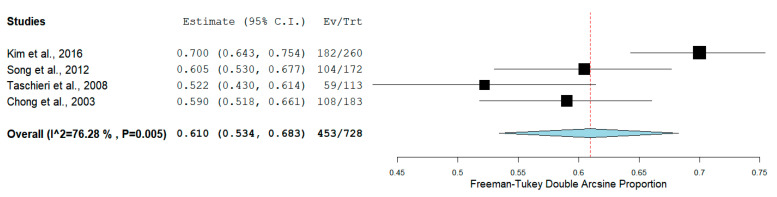
Forest plot of recall rate for randomized clinical trials (RCTs).

**Figure 4:**
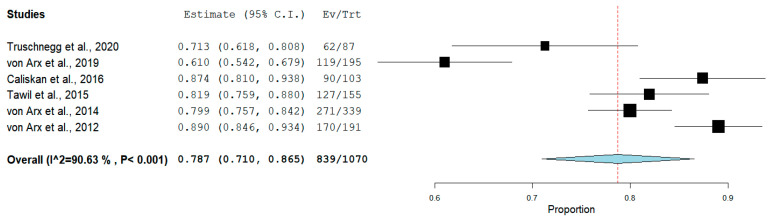
Forest plot of recall rate for prospective clinical studies (PCSs).

The pooled proportion of recall rate was 61.0% (95% CI = 53.4–68.3%, *p* < 0.001) for RCTs (Figure 3), while for PCSs was 78.7% (95% CI = 71.0–86.5%, *p* < 0.001) (Figure 4).

**Figure 5:**
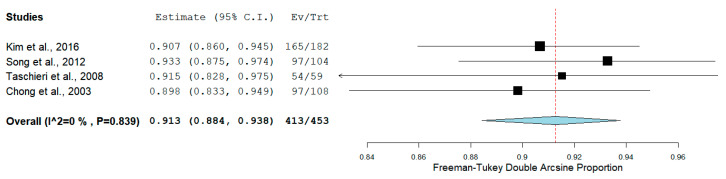
Forest plot of success rate outcome for randomized clinical trials (RCTs).

**Figure 6:**
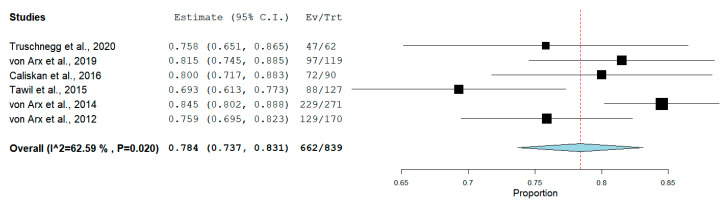
Forest plot of success rate outcome for prospective clinical studies (PCSs).

The pooled proportion of success rate, from an overall amount of 453 treated teeth, was 91.3% (95% CI = 88.4–93.8%, *p* < 0.001) for RCTs (Figure 5). From overall 839 included teeth in PCSs, a pooled success rate of 78.4% (95% CI = 73.7–83.1%, *p* < 0.001) (Figure 6) was observed.

Better prognosis was reported in two studies [7,32] for root-end filling with ProRoot MTA. Therefore, meta-analysis of the impact of root-end filling material on the prognosis was attempted. However, only two included RCT [11,39] and two PCS [7,32] reported data for this possible comparison, which was considered potentially misleading.

## 4. Discussion

The main objective of outcome assessment after endodontic treatment is to observe healing or improvement in apical periodontitis [42]. Based on the results of this review, 2 to 13 years after intervention, the pooled success rate of EMS ranged from 78% in PCS [2,7,32,35,36,37] to 91% pooled success rate of included RCT [9,11,38,39]. The overall success rate of EMS ranged from 69.3% [37] to 93.3% [38], showing a 24% difference between the minimum and the maximum clinical and radiographic healing observed among included studies. This wide value range might be explained by the methodological design of the studies. Tawil et al., 2015 [37], aimed to evaluate the post-surgical periapical healing response of roots with dentinal defects, diagnosed with the support of transillumination when compared with intact roots. Therefore, this study included a group of teeth with root dentinal defects, which had an extremely low success rate compared to the other group of evaluated teeth. In addition, the authors considered incomplete healed classified cases as non-healed. For these reasons, a significant decrease in the overall success rate was verified. On the other hand, Song et al., 2012 [38] just presented the outcome of the teeth considered as healed at the short-term follow-up (ranging from less than 1 year to 5 years) [43], which makes 39.5% its real recall rate, instead of 60.5%, this fact may possibly have led to an overestimation of the outcome results. The pooled recall rate of RCT studies included in this review was 61%, showing that a significant percentage of the inception cohort of patients was not available for follow-up, therefore, their treatment outcome is unknown [17], and the results may be skewed.

Between all the potential prognostic factors evaluated, only five presented statistically significant differences regarding the EMS outcome: smoking habits [35], tooth location and type (2); absence/presence of dentinal defect [37], interproximal bone level [7], and root-end filling material [7,32]. The impact of root-end filling material was the most frequently analyzed intraoperative factor among the included studies.

Modern endodontic approaches do not contemplate the use of amalgam as root-end filling material due to its potential disadvantages [44]; therefore, none of the included studies resorted to its use. Moreover, the use of gutta-percha alone or Glass Ionomer Cement (GIC) for EMS was not reported in these studies. In the present review, the root-end filling materials used for outcome evaluation were the following: Zinc oxide-eugenol intermediate restorative cement (IRM) [35,38,39], Super ethoxybenzoic acid (SuperEBA) [7,9,11,37,38], resin-based cements [7,32], and Mineral Trioxide Aggregate (MTA) [2,7,11,32,36,37,38,39].

Zinc oxide-eugenol (ZOE) is characterized by a combination of eugenol liquid and zinc oxide powder. To improve its mechanical properties, ZOE has been modified into other materials, for instance, Intermediate Restorative Material (IRM) and Super Ethoxybenzoic Acid (SuperEBA), without eugenol. Potential disadvantages of these cements have been described, such as tissue irritation, moisture sensitivity [38], high solubility, and challenging handling properties [44]. Furthermore, SuperEBA has a particular issue related to the possible development of air bubbles resulting in shrinkage when an inadequate powder-to-liquid proportion is used for the filling, which might cause microleakage in the long-term [38]. Chong et al., 2003 [39], Song et al., 2012 [38], and Truschnegg et al., 2020 [35] used IRM as root-end filling material. Chong et al., 2003 [39] was the only study showing comparative results on the EMS outcome between IRM and other material, even though the success rate was higher in the MTA group, no statistically significant difference was found. On the other hand, Taschieri et al., 2008 [9], von Arx et al., 2012 [7], Song et al., 2012 [38], Tawil et al., 2015 [37], and Kim et al., 2016 [11], used SuperEBA as retrograde filling material. von Arx et al., 2012 [7], was the only author who found statistically significant differences between the MTA group (86.4%) and the SuperEBA group (67.3%), with a higher success rate for MTA.

In the present study, there were two articles [7,32] that evaluated the EMS outcome when a dentine bonding agent was used. Both of them presented a lower success rate in comparison with MTA; however, only one [32] showed a statistically significant difference between the two root-end-filling materials. These results may be explained by the necessity of a dry field during the etch/prime/bond process [21], and the requirement of moisture control of such material [44], which can be difficult during the EMS procedure.

In the late 1990s, MTA, the first generation hydraulic calcium–silicate cements were introduced in dentistry [44], and later on, the second generation emerged with Biodentine, which improved some limitations of MTA, such as the induction of discoloration [45,46], the extended setting time, and the retarded hydration [47,48], through the replacement of bismuth oxide (Bi_2_O_3_) for zirconium oxide (ZrO_2_) as radiopacifier agent. These materials received widespread interest due to their high biocompatibility [49]. In the present study, most of the included studies used MTA as root-end filling material [2,7,11,32,36,37,38,39]. Indeed, MTA showed higher success values compared to SuperEBA [7] and COMP [32]; these outcome results can be supported by the characteristics described above. Even so, there are some clinical concerns regarding MTA, such as the probability of washing out because of its long setting time (2 h 45 min), and the fact of having a sandy consistency after its mix with sterile water, which makes it more difficult to handle, deliver to the operative site, and condense adequately [44].

Recently, new tricalcium silicate-based materials that maintain the desirable properties of prior bioceramic materials and overcome their disadvantages have been developed. The presence of calcium phosphate improves the setting properties of these materials, establishing a crystalline structure comparable with the tooth and bone apatite [50]. This type of material presents putty consistency and faster setting time, becoming easier to handle and deliver to the operative site [51]. However, the scientific evidence that supports its clinical use as a root-end filling material remains scarce. In fact, some studies reporting its use were excluded from our review, due to its short follow-up period of 1-year [6,8] and to its retrospective study design [52]. Despite their short follow-up periods, Safi et al. [6] and Zhou et al. [8] reported promising overall success rates evaluated through 2-dimensional periapical radiographies for root repair materials (RRM) of 92% and 94.4%, respectively.

With regard to quality assessment, the risk of bias was calculated for all studies included, either RCTs or PCSs. A low overall risk of bias was obtained for all studies, except Song et al. [38]. One of our greatest concerns is related to the risk of bias due to missing data. In fact, there were some studies that included extracted teeth in the statistical analysis [2,9,36]. However, there were several authors who considered teeth extracted during the follow-up as a dropout, because the reason for extraction was unknown or not related to EMS, such as fracture or prosthetic reasons [7,11,32]. Contemplating the patient-centered outcome, this information should not be disposable, since tooth retention is a major concern and missing extraction data will lead to an overestimation of the expected EMS outcome, independent of the reason for extraction. Another important concern is associated with the risk of bias due to measuring outcomes. Most of the studies had two or more observers [2,7,9,11,32,35,36,37,38,39], blinded [7,9], and an interobserver variability was assessed [2,11,36,38,39]. All studies used the radiographic outcome classification defined by Rud et al. [13] and Molven et al. [14]. Nevertheless, one study [37] classified incomplete healing cases as non-healed, which compromised this domain in the risk of bias evaluation. Indeed, an underestimation of the EMS outcome may have occurred, as explained above.

Concerning the follow-up period after endodontic microsurgery, the amount of time required for the outcome assessment remains questionable [1]. There is a need to overcome this particular issue to accomplish long-term predictability of the treated teeth and to make a weighted therapeutic decision [38].

The European Society of Endodontology (ESE) defines that regular clinical and radiographic follow-ups for a minimum observation period of 1-year are appropriate. However, longer periods may be necessary when complete healing is not accomplished or in other specific cases. Regarding the outcome assessment of surgical endodontic procedures, ESE specifies that when a radiolucent area, defined as “surgical defect” or “scar”, persists after 1-year, it must be monitored for the next 4 years [42]. According to the American Association of Endodontists (AAE), the outcome assessment should be performed for 1-year or beyond [53].

There is previous evidence reporting reversal to disease after 4 years following traditional endodontic surgery, which supports that a short follow-up period might not be enough to identify a recurrence of apical periodontitis [1]. However, such findings were not reported in studies with a modern microsurgical approach [11,37,39].

Recent long-term follow-up studies sought statistically significant differences in the outcome of EMS when long-term follow-up is compared to the outcome at a short-term follow-up period. Von Arx et al. [2] showed a significantly lower success rate after 10 years (81.5%) compared with the rates after 1 and 5 years (91.6% and 91.4%, respectively). However, no statistically significant differences were found when 1- and 5-years follow-up when compared. On the other hand, Kim et al. [11] demonstrated a slight reduction (4.8%) in the overall success rate at 4-year follow-up, but there was no statistically significant difference between 1- and 4-year follow-up periods. In fact, the authors attributed the lower recall rate of the 1-year follow-up of the success group at the 4-year follow-up as the main cause of this decrease. Two studies [37,39] showed that significant information about healing patterns was revealed 1-year after EMS. Furthermore, Von Arx et al. [32] confirmed that cases rated as healed after 1-year remained so in 93.9% of cases, after 5 years, with higher predictive value for MTA group (96.7%) in comparison with the COMP group (90.7%). Nevertheless, the classification of uncertain healing at a short-term follow-up appears to be the least predictable of all at a long-term follow-up [36,54].

Some studies suggest that the regression would possibly be counterbalanced by teeth that could be classified as healed at the long-term follow-up but were failures in the short-term [7,38].

For all the reasons described above, 1-year follow-up may be sufficient to estimate the predictability of EMS outcome at a long-term follow-up; nevertheless, uncertain healing cases at 1-year should continue to be carefully followed, and the root-end material used must be considered. On the other hand, long-term follow-up studies allow the achievement of a more reliable patient-centered outcome, due to the possibility of existence of data concerning the survival rate and increase the knowledge of the risk factors involved in long-term failures, such as root fracture [2], prosthodontic considerations [7], endodontic or periodontological reasons [35], and crown fractures or caries [11].

In this review, strict inclusion and exclusion criteria were employed, with the aim of decreasing the heterogeneity of the included studies and, consequently, achieve the most reliable results possible. Contrary to Tsesis et al. [19], articles whose surgical procedure was not performed under endoscope or microscope were excluded. Concerning the study design, some reviews [20,21] included retrospective cohort studies (RCSs), which was not permitted in this review according to the exclusion criteria. Finally, the last strength of this study is the fact that all studies used the same radiographic outcome classification [13,14], which allowed making an easier comparison of the outcome results.

Some limitations were found in this study. First, only studies with a long-term follow-up period were included, which compromised the quality of some of them, since the longer the follow-up, the higher the dropout rate [4,38], which might lead to an inherent loss of scientific validity of some conclusions [38]. Second, it was challenging to make an objective data interpretation due to the lack of criteria consistency among the studies; there is a need to establish guidelines to report outcomes targeted to studies on EMS. Lastly, despite the current trend of the search for comparative outcome results between 2-Dimensional (2D) and 3-Dimensional (3D) outcome measurement, the inclusion of such studies [55,56,57] was not possible due to a short follow-up time [57], a retrospective study design [56], or to the clinical and radiographic criteria applied [55]. Although there are studies reporting that 2D evaluation overestimates healing compared with 3D [23,55], Kruse et al. [18] developed a study with the aim to make a comparison of the diagnostic validity between 2D and 3D outcome measurement to determine the true nature of periapical lesions diagnosed through these two different radiographic methods. In fact, the authors reported that more than 40% of the unsuccessful classified cases diagnosed through CBCT did not show signs of periapical inflammation after histopathological examination. Therefore, it was concluded that prudence should be employed when CBCT is used, as this method may diagnose “scar tissue healing” (incomplete healing) as the presence of pathology (uncertain healing), underestimating healing. According to ESE [58], CBCT should only be used when its advantages exceed conventional imaging benefits. For the reason that there is a need to overcome the doubt about the nature of the lesions through CBCT evaluation, and a reliable healing assessment system based on CBCT is yet to be validated, so far, it is still judicious to conform with the ALARA principle (as low as reasonably achievable) which restricts the clinical use of CBCT for EMS follow-up on a case-by-case basis [58].

Several concerns were found regarding the external validity of the results achieved. First, all included studies were mainly carried out by specialists in a Hospital or University environment [2,7,9,11,32,35,36,37,38,39], which can lead to an overestimation of the outcome when the procedure is performed under a private clinical setting. Since the outcome has been reported to be influenced by the operator [54], there is a need to develop multicenter, pragmatic studies to evaluate the outcome of EMS in distinct conditions, as what happens on a daily basis in clinical practice. Furthermore, some authors established some rigorous exclusion criteria, such as teeth with a probing depth ≥4 mm [39], teeth that did not undergo NSER, or teeth with traumatic injuries [9]. All those criteria are focused on maximizing the efficacy of the intervention, which may contribute to overestimating the EMS outcome and, consequently, compromise the external validity of the results and transference of this evidence to everyday clinical practice (effectiveness of the intervention).

Lastly, no cost-benefit ratio regarding the root-end filling material was performed in any of the studies. This ratio might be interesting to consider in a therapeutic decision for both the dentist and the patient.

## 5. Conclusions

The long-term EMS outcome showed high pooled success rates, from 78 to 91% when followed-up from 2 to 13 years. This treatment approach is predictable when performed under modern surgical techniques and associated with biocompatible and bioactive root-end filling materials, allowing tooth survival ranging from 79 to 100%, 2 to 13 years after treatment. Moreover, EMS may be influenced by the following potential prognostic factors: smoking habits, tooth location and type, absence/presence of dentinal defect, interproximal bone level, and root-end filling material. Regarding the root-end filling material, ProRoot MTA showed a trend to better outcome results.

## Figures and Tables

**Table 1:** Studies included in the review and the success rate.

Study	Design	No. of Teeth (n)	Follow-Up (Years)	Root-End Filling Material	Recall Rate (%)	Success Rate for Each Root-End Filling	Overall Success Rate	Survival Rate *
Truschnegg et al., 2020 [35]	PCS	87	10 to 13	IRM	71% (62/87)	N.A.	76% (47/62)	79%
von Arx et al., 2019 [2]	PCS	195	10	ProRoot^®^ MTA grey (*n* = 44)	61% (119/195)	ProRoot^®^ MTA grey 84% (37/44)	82% (97/119)	88%
				ProRoot^®^ MTA white (*n* = 75)		ProRoot^®^ MTA white 80% (60/75)		
Kim et al., 2016 [11]	RCT	260	4	ProRoot^®^ MTA (*n* = 83)	70% (182/260)	ProRoot^®^ MTA 92% (76/83)	91% (165/182)	92%
				Super EBA (*n* = 99)		Super EBA 90% (89/99)		
Caliskan et al., 2016 [36]	PCS	103	2 to 6	ProRoot^®^ MTA (*n* = 90)	87% (90/103)	N.A.	80% (72/90)	100%
Tawil et al., 2015 [37]	PCS	155	3	ProRoot^®^ MTA grey or SuperEBA (*n* = 127)	82% (127/155)	N.A.	69% (88/127)	100%
von Arx et al., 2014 [32]	PCS	339	5	ProRoot MTA^®^ (*n* = 134)	80% (271/339)	ProRoot MTA^®^ 93% (124/134)	85% (229/271)	89%
				COMP (*n* = 137)		COMP 77% (105/137)		
Song et al., 2012 [38]	RCT	172	6 to 10	IRM, Super EBA or ProRoot^®^ MTA (*n* = 104)	61% (104/172)	N.A.	93% (97/104)	100%
von Arx et al., 2012 [7]	PCS	191	5	ProRoot MTA^®^ (*n* = 44)	88% (170/191)	ProRoot MTA^®^ 86% (38/44)	76% (129/170)	93%
				SuperEBA (*n* = 49)		SuperEBA 67% (33/49)		
				Retroplast (*n* = 77)		Retroplast 75% (58/77)		
Taschieri et al., 2008 [9]	RCT	113	2	SuperEBA (*n* = 59)	89% (59/113)	N.A.	92% (54/59)	100%
Chong et al., 2003 [39]	RCT	183	2	MTA (*n* = 61)	59% (108/183)	MTA 92% (56/61)	90% (97/108)	100%
				IRM (*n* = 47)		IRM 87% (41/47)		

Prospective clinical study (PCS), Randomized clinical trial (RCT), not available (N.A.), zinc oxide-eugenol intermediate restorative cement (IRM), mineral trioxide aggregate (MTA), dentin-bonded adhesive resin composite (COMP), Super ethoxybenzoic acid (SuperEBA). * rate not reported in the articles, calculated based on available values.

**Table 2:** Data summary of the included studies analysis.

Study	Study Design	Material	Evaluated Parameters	Follow-up (years)	Sample Size		Previous Treatments		Success Rate (%)			Recall Rate (%)	Results: Prognostic Factors
					No. Patients	No. Teeth	No. Re-Surgery	No. NSER	Clinical	Radiographic			
										Healed	Non-Healed		
Truschnegg et al., 2020 [35]	PCS	IRM	1. Age 2. Sex 3. Smoking and alcohol habits 4. Tooth location 5. Previous endodontic surgery 6. Size of the pre and postoperative lesion 7. Perioperative antibiotics	10 to 13	73	87	19	0	79	76	24	71	No significant differences: Age, sex, alcohol habits, tooth location, previous endodontic surgery, size of the pre and postoperative lesion, or perioperative antibiotics. Significant differences: Smokers (lower success rate).
von Arx et al., 2019 [2]	PCS	ProRoot^®^ MTA Grey or ProRoot^®^ MTA White	1. Sex 2. Age 3. Tooth type 4. Type of ProRoot^®^ MTA used (grey vs. white) 5. Surgery (first-time vs. repeat surgery)	10	NA	119	12	NA	NA	Overall rate: 82 MTA grey Group: 84 MTA white Group: 80	Overall rate: 18 MTA grey Group: 16 MTA white Group: 20	61	No significant differences: Age, sex, type of MTA, or first-time versus repeat surgery. Significant differences: Tooth type (Higher success rate for maxillary molars compared to maxillary premolars).
Kim et al., 2016 [11]	RCT	ProRoot^®^ MTA, Super EBA	1. Type of material	4	NA	260	NA	NA	NA	Overall rate: 91 ProRoot^®^ MTA Group: 92 SuperEBA Group: 90	Overall rate: 9 ProRoot^®^ MTA Group: 8 SuperEBA Group: 10	70	No significant differences: Type of material.
Caliskan et al., 2016 [36]	PCS	ProRoot^®^ MTA	1. Sex 2. Age 3. Tooth location and type 4. Quality of the root canal filling 5. Presence or absence of a post6. Previous endodontic treatment or retreatment 7. Previous nonsurgical or surgical endodontic treatment 8. Size and histopathology of periapical lesions 9. Antibiotic therapy 10. Postoperative healing	2 to 6	108	108	18	42	NA	80	20	87	No significant differences: Sex, age, tooth location and type, quality of the root canal filling, presence or absence of a post, previous endodontic treatment or retreatment, previous nonsurgical or surgical endodontic treatment, size and histopathology of periapical lesions, antibiotic therapy, or postoperative healing course.
Tawil et al., 2015 [37]	PCS	ProRoot^®^ MTA grey or SuperEBA	1. Sex 2. Age 3. Tooth location 4. Presence vs. absence of dentinal defect 5. Root-end filling material (Super EBA vs. MTA)	3	NA	155	NA	NA	NA	Overall rate: 69 Dentinal defect Group: 32 Intact Group: 97	Overall rate: 31 Dentinal defect Group: 68 Intact Group: 3	82	No significant differences: Sex, Age, Tooth location, or root-end filling material (Super EBA vs. MTA) Significant differences: Presence of dentinal defect (lower success rate)
von Arx et al., 2014 [32]	PCS	ProRoot^®^ MTA, dentin-bonded adhesive resin composite (COMP)	1. Type of material (MTA or COMP) 2. Age 3. Sex 4. Tooth type (maxillary anterior, premolar, and molar or mandibular anterior, premolar, and molar) 5. Presence or absence of post/screw 6. Type of surgery (first-time surgery or repeat surgery).	5	339	339	31	NA	NA	Overall rate:85 MTA Group: 93 COMP Group:77	Overall rate:15 MTA Group: 7 COMP Group: 23	80	No significant differences: Age, sex, type of tooth treated, presence of post/screw, or type of surgery (first-time vs. repeated surgery). Significant differences: Type of material (higher success rate for MTA treated teeth)
Song et al., 2012 [38]	RCT	IRM, Super EBA, ProRoot^®^ MTA	1. Age 2. Sex 3. Tooth type 4. Tooth location 5. Lesion type (A-F) 6. Type of material	6 to 10	NA	172	NA	NA	NA	93	7	61	NA
von Arx et al., 2012 [7]	PCS	SuperEBA, ProRoot^®^ MTA, Retroplast	1. Patient-related (i.e., age, sex, and smoking) 2. Tooth related (i.e., tooth type, pain, clinical signs/symptoms, size of periapical lesion, interproximal bone level, apical extent of root canal filling, post, and previous apical surgery) 3. Treatment related (i.e., antibiotic prescription, root-end filling material, and initial postoperative healing)	5	194	194	16	NA	85	Overall rate:76 ProRoot^®^ MTA Group: 88 SuperEBA Group: 67 Retroplast Group: 75	Overall rate: 24 ProRoot^®^ MTA Group: 12SuperEBA Group: 33 Retroplast Group: 25	88	No significant differences: Patient related factors, tooth related factors (i.e., tooth type, pain, clinical signs/symptoms, size of periapical lesion, apical extent of root canal filling, post, and previous apical surgery), or treatment related factors (i.e., antibiotic prescription, and initial postoperative healing). Significant differences: Interproximal bone level (higher success rate when the mesial and distal interproximal bone level was ≤ 3 mm from the cementoenamel junction (or restoration margin), or type of material (higher success rate for ProRoot^®^ MTA when compared to SuperEBA).
Taschieri et al., 2008 [9]	RCT	SuperEBA	1. Type of magnification device (microscope vs. endoscope) 2. Tooth location	2	70	113	NA	113	NA	92 Zinc-oxide EBA-reinforced cement	8 Zinc-oxide EBA-reinforced cement	89	No significant differences: Type of magnification device (microscope vs. endoscope), or tooth location (arch)
Chong et al., 2003 [39]	RCT	MTA (developed at Loma Linda University, California), IRM	1. Type of material (MTA or IRM)	2	183	183	NA	NA	NA	Overall rate: 90 MTA Group: 92 IRM Group: 87	Overall rate:10 MTA Group: 8 IRM Group: 13	59	No significant differences: Type of material

Prospective clinical study (PCS), Randomized clinical trial (RCT), not available (N.A.), zinc oxide-eugenol intermediate restorative cement (IRM), mineral trioxide aggregate (MTA), dentin-bonded adhesive resin composite (COMP), Super ethoxybenzoic acid (SuperEBA).

**Table 3:** Risk of bias summary of the included prospective clinical studies (PCSs).

	Domains							
	Pre intervention		At intervention	Post intervention				
**Study**	Bias due to Confounding	Bias in Selecting Participants for the Study	Bias in Classifying Interventions	Bias due to Deviations from Intended Intervention	Bias due to Missing Data	Bias in Measuring Outcomes	Bias in Selecting Reported Result	Overall RcB Judgment
**Truschnegg et al., 2020 [35]**	Moderate	Low	Low	Low	Low	Low	Low	Low
**von Arx et al., 2019 [2]**	Low	Low	Low	Low	Low	Low	Low	Low
**Caliskan et al., 2016 [36]**	Moderate	Low	Low	Low	Low	Moderate	Low	Low
**Tawil et al., 2015 [37]**	Low	Low	Low	Low	Low	Moderate	Moderate	Low
**von Arx et al., 2014 [32]**	Low	Low	Low	Low	Low	Moderate	Low	Low
**von Arx et al., 2012 [7]**	Low	Low	Low	Low	Low	Low	Low	Low

## Supplementary Materials

The following are available online at https://www.mdpi.com/1010-660X/56/9/447/s1, Table S1: Radiographic outcome criteria for EMS described by Rud et al. and Molven et al., Supplementary S1: Pubmed electronic database search strategy, Supplementary S2: The Cochrane Library electronic database search strategy, Table S2: Reasons for articles exclusion according to the criteria previously established.
