# Supplementary material for: Long-Term Prognosis of Endodontic Microsurgery—A Systematic Review and Meta-Analysis
Source: Medicina (Kaunas). 2020 Sep 3;56(9):447. doi: 10.3390/medicina56090447 (PMC7558840; doi:10.3390/medicina56090447)
Supplement: Supplementary file 1 [file medicina-56-00447-s001.pdf]

Supplementary materials

Table S1: Radiographic outcome criteria for EMS described by Rud *et al* and Molven *et al*.

**Complete Healing***	1. Re-formation of periodontal space of normal width and lamina dura to be followed around the apex.<br>2. Slight increase in width of apical periodontal space, but less than twice the width of non-involved parts of the root.<br>3. Tiny defect in the lamina dura (maximum 1mm) adjacent to the root filling.<br>4. Complete bone repair; bone bordering the apical area does not have the same density as surrounding non-involved bone.<br>5. Complete bone repair; no apical periodontal space can be discerned.
**Incomplete Healing***	The rarefaction has decreased in size or remained stationary, and is characterized by:<br>1. Bone structures are recognized within the rarefaction; the periphery of the rarefaction is irregular and may be demarcated by a compact bone border; the rarefaction is located asymmetrically around the apex; the connection of the rarefaction with the periodontal space is angular;<br>2. Isolated scar tissue in the bone.
**Uncertain Healing****	The rarefaction has decreased in size, and with one or more of the following characteristics:<br>1. The RL is larger than twice the width of periodontal space.

	2. The RL is bordered by lamina-dura like bone structures.<br>3. The RL has a circular or semicircular periphery.<br>4. The RL is located symmetrically around the apex as a funnel-shaped extension of the periodontal space.
**Unsatisfactory Healing****	The RL area appears enlarged or unchanged.

*Complete and incomplete/scar categories were combined as success.

** Uncertain and unsatisfactory healing were combined as failure.

**Exclusion criteria**	**Articles**
**2**	Song *et al.*, 2018; Al-Nuaimi *et al.*, 2018; Kruse *et al.*, 2017; Kim *et al.*, 2016; da Silva *et al.*, 2015; Shinbori *et al.*, 2015; Mente *et al.*, 2015; Kang *et al.*, 2015; Lui *et al.*, 2014; Song *et al.*, 2014; Bryce *et al.*, 2013; Angiero *et al.*, 2011; Taschieri *et al.*, 2011; Moshonov *et al.*, 2011; Song *et al.*, 2011; Taschieri *et al.*, 2010; Salehrabi *et al.*, 2010; Gilbert *et al.*, 2010; Tsesis *et al.*, 2009; Jonasson *et al.*, 2008; Iqbal *et al.*, 2007; Oberli *et al.*, 2007; Xu *et al.*, 2006; Tsesis *et al.*, 2006; von Arx *et al.*, 2005; Oginni *et al.*, 2002; Rahbaran *et al.*, 2001; Testori *et al.*, 1999; Danin *et al.*, 1999; Rud *et al.*, 1998; Mor *et al.*, 1995
**3**	Kruse *et al.*, 2017; Kacarska *et al.*, 2017; Wang *et al.*, 2017; Kruse *et al.*, 2016; Song *et al.*, 2013; Saunders *et al.*, 2008; Wang *et al.*, 2004
**4**	Kulakov *et al.*, 2018; Kruse *et al.*, 2017; Kacarska *et al.*, 2017; Barone *et al.*, 2010; Penarrocha *et al.*, 2007; Yazdi *et al.*, 2007; Leco Berrocal *et al.*, 2007; Gagliani *et al.*, 2005; Maddalone *et al.*, 2003; Rud *et al.*, 2001; Zuolo *et al.*, 2000; Kvist *et al.*, 1999; Rud *et al.*, 1996; Sumi *et al.*, 1996; Molven *et al.*, 1996; Rud *et al.*, 1996; Jesslen *et al.*, 1995
**5**	Safi *et al.*, 2019; Penarrocha *et al.*, 2019; Meschi *et al.*, 2018; Zhou *et al.*, 2017; Kacarska *et al.*, 2017; von Arx *et al.*, 2016; von Arx *et al.*, 2016; von Arx *et al.*, 2016; Jorge *et al.*, 2015; Kurt *et al.*, 2014; Song *et al.*, 2013; Kreisler *et al.*, 2013; Song *et al.*, 2012; Shen *et al.*, 2012; Patel *et al.*, 2012; Walivaara *et al.*, 2011; von Arx *et al.*, 2010; Walivaara *et al.*, 2009; Shearer *et al.*, 2009; Taschieri *et al.*, 2008; Carrillo *et al.*, 2008; Garcia *et al.*, 2008; Kim *et al.*, 2008; Penarrocha *et al.*, 2008; Taschieri *et al.*, 2007; de Lange *et al.*, 2007; Penarrocha *et al.*, 2007; Walivaara *et al.*, 2007; von Arx *et al.*, 2007; Taschieri *et al.*, 2007; Taschieri *et al.*, 2006; Marin-Botero *et al.*, 2006; Filippi *et al.*, 2006; Taschieri *et al.*, 2005; Marti-Bowen et al, 2005; von Arx *et al.*, 2003; Dietrich *et al.*, 2003; Jensen *et al.*, 2002; Vallecillo Capilla *et al.*, 2002; von Arx *et al.*, 2001; von Arx *et al.*, 1999; Danin *et al.*, 1996; Van Doorne *et al.*, 1996; Pecora *et al.*, 1995
**6**	von Arx *et al.*, 2019; Kulakov *et al.*, 2018; Kacarska *et al.*, 2017

**7**	von Arx *et al.*, 2019; Penarrocha *et al.*, 2019; Riis *et al.*, 2018; Meschi *et al.*, 2018; Kulakov *et al.*, 2018; Kacarska *et al.*, 2017; Menendez-Nieto *et al.*, 2016; Caliskan *et al.*, 2016; Taschieri *et al.*, 2014; von Arx *et al.*, 2011; Wang *et al.*, 2004; Wang *et al.*, 2004
**8**	Zandi *et al.*, 2019; Castro *et al.*, 2018; Meschi *et al.*, 2018; Kulakov *et al.*, 2018; Prati *et al.*, 2018; Fariniuk *et al.*, 2017; Kruse *et al.*, 2017; Kacarska *et al.*, 2017; Menendez-Nieto *et al.*, 2016; Neskovic *et al.*, 2016; Patel *et al.*, 2012; Song *et al.*, 2011; de Chevigny *et al.*, 2008; Xu *et al.*, 2006; Farzaneh *et al.*, 2004; Sjogren *et al.*, 1997

Table S2: Reasons for articles exclusion according to the criteria previously established.

Supplementary S1: Pubmed electronic database search strategy.

The term: "(((((((periapical diseases[MeSH Terms]) OR periapical diseases) OR root-end filling) OR root canal therapy[MeSH Terms]) OR root canal therapy)) AND ((((((((((((((apicoectomy[MeSH Terms]) OR apicoectomy) OR surgical endodontic retreatment) OR apical surgery) OR retreatment[MeSH Terms]) OR apical surgery) OR periapical surgery) OR retrograde surgery) OR endodontic surgery) OR root-end surgery) OR root-end cavity preparation) OR periradicular surgery) OR microsurgery[MeSH Terms]) OR retreatment[MeSH Terms]) OR root-end ressection) OR apicectomy)) AND (((((radiographic outcome) OR treatment outcome[MeSH Terms]) OR treatment outcome) OR success rate) OR radiographic success rate) NOT (review OR case report)" was used on Pubmed search. Limits used were studies on humans published from 1990/01/01.

Supplementary S2: The Cochrane Library electronic database search strategy.

The term: "[((Retrograde Obturation [MeSH descriptor]) OR (retrograde obturation) OR (root end filling)) AND ((Apicoectomy[MeSH descriptor]) OR (apicoectomy) OR (apical surgery) OR (periapical surgery) OR (Microsurgery[MeSH descriptor]) AND ((Treatment Outcome[MeSH descriptor]) OR (success rate) OR (radiographic success rate))]" was used on The Cochrane Library search. Limits used were trials and studies published from 1990/01/01.
